# Supplementary material for: Resolving the full spectrum of human genome variation using Linked-Reads
Source: Genome Res. 2019 Apr;29(4):635–45. doi: 10.1101/gr.234443.118 (PMC6442396; doi:10.1101/gr.234443.118)
Supplement: Supplemental Material [file supp_gr.234443.118_Supplemental_longranger-2.2.zip › longranger-2.2.tar.bz2!/lr2.2-public/lib/stan/cmdstan/stan/doc/api/html/dirichlet_8hpp_source.html]

Stan: src/stan/prob/distributions/multivariate/continuous/dirichlet.hpp Source File


|  |  |
| --- | --- |
|  | Stan  2.5.0  probability, sampling & optimization |


- Main Page
- Related Pages
- Namespaces
- Classes
- Files

- File List
- File Members

All Classes Namespaces Files Functions Variables Typedefs Enumerations Enumerator Friends Macros Pages

- src
- stan
- prob
- distributions
- multivariate
- continuous

dirichlet.hpp

Go to the documentation of this file.

1 #ifndef STAN\_\_PROB\_\_DISTRIBUTIONS\_\_MULTIVARIATE\_\_CONTINUOUS\_\_DIRICHLET\_HPP

2 #define STAN\_\_PROB\_\_DISTRIBUTIONS\_\_MULTIVARIATE\_\_CONTINUOUS\_\_DIRICHLET\_HPP

3

4 #include <boost/math/special\_functions/gamma.hpp>

5 #include <boost/random/gamma\_distribution.hpp>

6 #include <boost/random/variate\_generator.hpp>

7

8 #include <stan/prob/constants.hpp>

9 #include <stan/math/matrix\_error\_handling.hpp>

10 #include <stan/math/error\_handling.hpp>

11 #include <stan/prob/traits.hpp>

12 #include <stan/math/functions/multiply\_log.hpp>

13

14 namespace stan {

15

16  namespace prob {

17

43  template <bool propto,

44  typename T\_prob, typename T\_prior\_sample\_size>

45  typename boost::math::tools::promote\_args<T\_prob,T\_prior\_sample\_size>::type

46  dirichlet\_log(const Eigen::Matrix<T\_prob,Eigen::Dynamic,1>& theta,

47  const Eigen::Matrix<T\_prior\_sample\_size,Eigen::Dynamic,1>& alpha) {

48  static const char\* function = "stan::prob::dirichlet\_log(%1%)";

49  using boost::math::lgamma;

50  using boost::math::tools::promote\_args;

51  using stan::math::check\_consistent\_sizes;

52  using stan::math::check\_positive;

53  using stan::math::check\_simplex;

54  using stan::math::multiply\_log;

55

56  typename promote\_args<T\_prob,T\_prior\_sample\_size>::type lp(0.0);

57  check\_consistent\_sizes(function, theta, alpha,

58  "probabilities", "prior sample sizes",

59  &lp);

60  check\_positive(function, alpha, "prior sample sizes", &lp);

61  check\_simplex(function, theta, "probabilities", &lp);

62

63  if (include\_summand<propto,T\_prior\_sample\_size>::value) {

64  lp += lgamma(alpha.sum());

65  for (int k = 0; k < alpha.rows(); ++k)

66  lp -= lgamma(alpha[k]);

67  }

68  if (include\_summand<propto,T\_prob,T\_prior\_sample\_size>::value)

69  for (int k = 0; k < theta.rows(); ++k)

70  lp += multiply\_log(alpha[k]-1, theta[k]);

71  return lp;

72  }

73

74  template <typename T\_prob, typename T\_prior\_sample\_size>

75  inline

76  typename boost::math::tools::promote\_args<T\_prob,T\_prior\_sample\_size>::type

77  dirichlet\_log(const Eigen::Matrix<T\_prob,Eigen::Dynamic,1>& theta,

78  const Eigen::Matrix<T\_prior\_sample\_size,Eigen::Dynamic,1>& alpha) {

79  return dirichlet\_log<false>(theta,alpha);

80  }

81

82  template <class RNG>

83  inline Eigen::VectorXd

84  dirichlet\_rng(const Eigen::Matrix<double,Eigen::Dynamic,1>& alpha,

85  RNG& rng) {

86  using boost::variate\_generator;

87  using boost::gamma\_distribution;

88

89  double sum = 0;

90  Eigen::VectorXd y(alpha.rows());

91  for(int i = 0; i < alpha.rows(); i++) {

92  variate\_generator<RNG&, gamma\_distribution<> >

93  gamma\_rng(rng, gamma\_distribution<>(alpha(i,0),1));

94  y(i) = gamma\_rng();

95  sum += y(i);

96  }

97

98  for(int i = 0; i < alpha.rows(); i++)

99  y(i) /= sum;

100  return y;

101  }

102  }

103 }

104 #endif

multiply\_log.hpp

traits.hpp

error\_handling.hpp

stan::math::multiply\_log

boost::math::tools::promote\_args< T\_a, T\_b >::type multiply\_log(const T\_a a, const T\_b b)

Calculated the value of the first argument times log of the second argument while behaving properly w...

**Definition:** multiply\_log.hpp:51

stan::math::check\_simplex

bool check\_simplex(const char \*function, const Eigen::Matrix< T\_prob, Eigen::Dynamic, 1 > &theta, const char \*name, T\_result \*result)

Return true if the specified vector is simplex.

**Definition:** check\_simplex.hpp:32

matrix\_error\_handling.hpp

constants.hpp

stan::agrad::sum

fvar< T > sum(const Eigen::Matrix< fvar< T >, R, C > &m)

**Definition:** sum.hpp:14

stan::agrad::lgamma

fvar< T > lgamma(const fvar< T > &x)

**Definition:** lgamma.hpp:15

stan::prob::include\_summand

Template metaprogram to calculate whether a summand needs to be included in a proportional (log) prob...

**Definition:** traits.hpp:35

stan::prob::dirichlet\_rng

Eigen::VectorXd dirichlet\_rng(const Eigen::Matrix< double, Eigen::Dynamic, 1 > &alpha, RNG &rng)

**Definition:** dirichlet.hpp:84

stan::math::check\_consistent\_sizes

bool check\_consistent\_sizes(const char \*function, const T1 &x1, const T2 &x2, const char \*name1, const char \*name2, T\_result \*result)

**Definition:** check\_consistent\_sizes.hpp:13

stan::math::check\_positive

bool check\_positive(const char \*function, const T\_y &y, const char \*name, T\_result \*result)

**Definition:** check\_positive.hpp:54

stan::agrad::multiply\_log

fvar< T > multiply\_log(const fvar< T > &x1, const fvar< T > &x2)

**Definition:** multiply\_log.hpp:15

stan::prob::gamma\_rng

double gamma\_rng(const double alpha, const double beta, RNG &rng)

**Definition:** gamma.hpp:491

stan::prob::dirichlet\_log

boost::math::tools::promote\_args< T\_prob, T\_prior\_sample\_size >::type dirichlet\_log(const Eigen::Matrix< T\_prob, Eigen::Dynamic, 1 > &theta, const Eigen::Matrix< T\_prior\_sample\_size, Eigen::Dynamic, 1 > &alpha)

The log of the Dirichlet density for the given theta and a vector of prior sample sizes...

**Definition:** dirichlet.hpp:46


---

[ Stan Home Page ]

*© 2011–2014,
Stan Development Team.*
